# Advances in neglected tropical disease vaccines: Developing relative potency and functional assays for the *Na*-GST-1/Alhydrogel hookworm vaccine

**DOI:** 10.1371/journal.pntd.0005385

**Published:** 2017-02-13

**Authors:** Jill B. Brelsford, Jordan L. Plieskatt, Anna Yakovleva, Amar Jariwala, Brian P. Keegan, Jin Peng, Pengjun Xia, Guangzhao Li, Doreen Campbell, Maria Victoria Periago, Rodrigo Correa-Oliveira, Maria Elena Bottazzi, Peter J. Hotez, David Diemert, Jeffrey M. Bethony

**Affiliations:** 1 Department of Microbiology, Immunology and Tropical Medicine, School of Medicine and Health Sciences, The George Washington University, Washington DC, United States of America; 2 Department of Pathology, School of Medicine and Health Sciences, The George Washington University, Washington DC, United States of America; 3 Department of Pediatrics, Section of Pediatric Tropical Medicine, Sabin Vaccine Institute and Texas Children's Hospital Center for Vaccine Development, Baylor College of Medicine, Houston, TX, United States of America; 4 Fundación Mundo Sano, Buenos Aires, Argentina; 5 Instituto René Rachou, Fundação Oswaldo Cruz, Belo Horizonte, Minas Gerais, Brazil; 6 National School of Tropical Medicine, Baylor College of Medicine, Houston, TX, United States of America; Ben-Gurion University of the Negev, ISRAEL

## Abstract

A new generation of vaccines for the neglected tropical diseases (NTDs) have now advanced into clinical development, with the *Na*-GST-1/Alhydrogel Hookworm Vaccine already being tested in Phase 1 studies in healthy adults. The current manuscript focuses on the often overlooked critical aspects of NTD vaccine product development, more specifically, vaccine stability testing programs. A key measure of vaccine stability testing is "relative potency" or the immunogenicity of the vaccine during storage. As with most NTD vaccines, the *Na*-GST-1/Alhydrogel Hookworm Vaccine was not developed by attenuation or inactivation of the pathogen (*Necator americanus*), so conventional methods for measuring relative potency are not relevant for this investigational product. Herein, we describe a novel relative potency testing program and report for the first time on the clinical lot of this NTD vaccine during its first 60 months of storage at 2–8°C. We also describe the development of a complementary functional assay that measures the ability of IgG from animals or humans immunized with *Na*-GST-1/Alhydrogel to neutralize this important hookworm enzyme. While 90% inhibition of the catalytic activity of *Na*-GST-1 was achieved in animals immunized with *Na*-GST-1/Alhydrogel, lower levels of inhibition were observed in immunized humans. Moreover, anti-*Na*-GST-1 antibodies from volunteers in non-hookworm endemic areas were better able to inhibit catalytic activity than anti-*Na*-GST-1 antibodies from volunteers resident in hookworm endemic areas. The results described herein provide the critical tools for the product development of NTD vaccines.

## Author summary

As vaccines targeting NTDs advance into clinical trials, product development and vaccine maintenance become critical activities for the success of these vaccines. A key activity during this phase of vaccine development is the “relative potency” of a vaccine or the quality of the immune response that the vaccine elicits in an animal model during storage to ensure its immunogenicity is maintained. As with most NTD vaccines, the *Na*-GST-1/Alhydrogel Hookworm Vaccine was not developed using traditional methods of attenuating the pathogen (*Necator americanus*), so traditional measures of relative potency, such as testing the vaccine’s ability to protect against lethal challenge, could not be used. For the first time, we describe the development of a relative potency testing program for an NTD vaccine during five years of storage at 2–8°C. We also describe the development of a complementary functional assay that measures the ability of IgG from animals or humans immunized with *Na*-GST-1/Alhydrogel to neutralize this important hookworm enzyme. The results described herein provide, for the first time in an open access format, critical tools for the development of future NTD vaccines.

## Introduction

Over the next decade, a new generation of vaccines for the neglected tropical diseases (NTDs), especially those for helminthic parasites such schistosomiasis and hookworm (*Necator americanus*) will advance into clinical trials [1]. The *Na*-GST-1/Alhydrogel Hookworm Vaccine has already entered into the initial stages of clinical development. As with other experimental vaccines for tropical diseases, including recombinant vaccines for *Plasmodium falciparum* malaria [2, 3], the *Na*-GST-1/Alhydrogel Human Hookworm Vaccine was not developed using the conventional methods of attenuation or inactivation of the pathogen that induce sterilizing immunity in the host [1]. The aim of the *Na*-GST-1/Alhydrogel Hookworm Vaccine is to induce neutralizing antibodies that will interfere with the role of glutathione S-transferase-1 (GST-1) in heme detoxification following blood digestion [4–6], inducing parasite death or reducing worm fecundity, thereby reducing protracted morbidity in the host [1, 7–10]. Conventional vaccine potency testing is often characterized by defining the doses of the vaccine that reproducibly protect immunized animals against lethal challenge from a pathogen and is in use for a number of vaccines, including those for tetanus, rabies, diphtheria, pertussis, and clostridia [11–14]. However, conventional vaccine potency testing programs are not feasible for the *Na*-GST-1/Alhydrogel Hookworm Vaccine due to the following characteristics of hookworms: (i) pathogenesis that results in chronic debilitating morbidity and not lethality, (ii) clinical outcomes that take years (sometimes even decades) to manifest in human hosts, and (iii) vaccine outcomes that are nearly impossible to replicate in laboratory animal models such as iron deficiency anemia from chronic hookworm infection as the animals become “refractory” to long-term infection [1, 15]. The vaccine potency testing program presented here is based on the levels of antibodies detected in the sera of animals immunized with defined doses of *Na*-GST-1/Alhydrogel [15]. Herein, we describe assessing the potency of the *Na*-GST-1/Alhydrogel Hookworm Vaccine formulation immediately after its manufacture (“potency at lot release”) and then its stability during 60 months of storage at 2–8°C (“relative potency”). This vaccine potency assay program can serve as a model for the expanding NTD vaccine community and aid in the product and clinical development of the next generation of recombinant NTD vaccines.

## Material and methods

### *Na*-GST-1/Alhydrogel hookworm vaccine

*Necator americanus* glutathione-S-transferase-1 (*Na*-GST-1) is a 24-kDa recombinant protein from *N*. *americanus* expressed in *Pichia pastoris* and purified by three chromatographic steps [16]. The *Na*-GST-1 Clinical Drug Product (Aeras Lot# 09-69F-001) was formulated at a concentration of 0.1 mg/mL *Na*-GST-1 with 0.8 mg/mL of Alhydrogel in a glucose/imidazole buffer (10% dextrose, 10mM imidazole, pH 7.4). The drug product (vaccine) was produced according to current Good Manufacturing Practices (cGMP) on November 17^th^, 2010 (Aeras, Rockville, MD) and thereafter stored in temperature monitored refrigerators at 2–8°C.

### Bioassay

*Vivarium*: Seven to eight-week-old BALB/c mice were obtained from Taconic (Gaithersburg, MD). Mice were housed in a Bio-safety level-2 animal facility with a room temperature of 72 ± 4°F under negative air pressure and with the air changed every 15–18 hours. A 12-hour day and dark light cycle was maintained in the animal facilities and the mice fed with Teklad Rodent Diet #2018. Mice were identified by ear puncture, and housed five animals per cage. Mice were acclimated to housing conditions one week prior to the start of the study. Mice were observed following immunizations for any signs of ill health (e.g., hunched, scruffy appearance, etc.). The animal studies reported herein were conducted at The George Washington University (Washington, DC), with the approval of its Institutional Animal Care and Use Committee (Protocol A042) and at the Baylor College of Medicine (Houston, TX) in compliance with its Institutional Animal Care and Use Committee (Protocol AN-5765).

**Fig 1:**
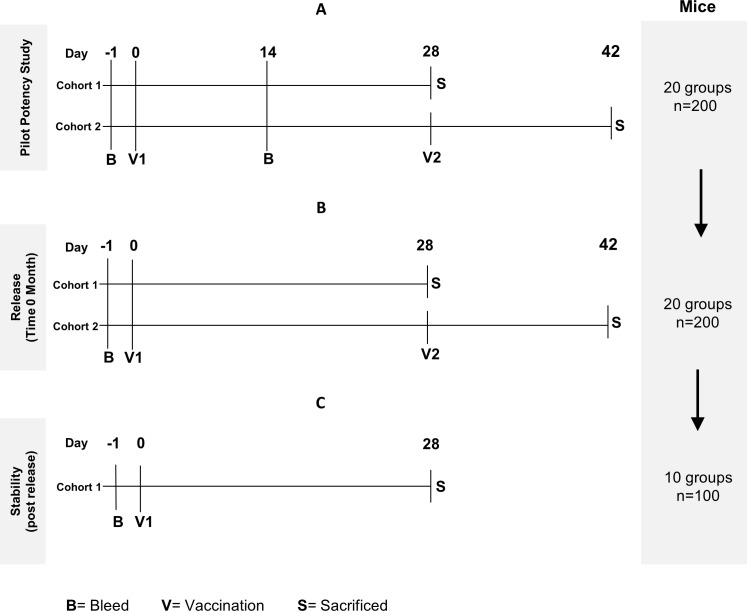
Vaccination and bleed schedules of the animal studies performed as part of the potency assay for *Na*-GST-1/Alhydrogel. The symbols are as follows: bleed (B), vaccination (V), and sacrifice (S). **(A)** The schedule of the pilot potency study with two cohorts, each consisting of ten dose groups of ten mice. **(B)** The schedule for potency at release of cGMP lot #09-69F-001 (T = 0 months). Two cohorts were used for this study, each consisting of ten groups of ten mice. Note: The IgG ELISA results of the pilot potency study (Panel A) showed that the levels of antibodies on Study Day 14 were low so this bleed time point was not performed in subsequent studies. **(C)** The schedule of the relative potency studies at 3, 6, 9, 12, 18, 24, 36, 48, and 60 months post manufacture. All mice were vaccinated intraperitoneally on Study Day 0 and bled on Study Days -1 and 28 in this final study design.

*Study Design*: Fig 1 shows the bioassay used for the determination of potency immediately after manufacturing (lot release) and relative potency over 60 months. The pilot dose-ranging study (Fig 1A) utilized research grade recombinant *Na*-GST-1, and was performed prior to release of the clinical lot. The pilot dose-ranging study and subsequent release study consisted of two immunization cohorts (Fig 1A,B): Cohort 1 was immunized using a prime-only regimen with a terminal bleed on Study Day 28; Cohort 2, was immunized with a prime-boost regimen (Day 0 and Day 28) followed by a terminal bleed on Study Day 42. The study designs used for determination of relative potency over the 60-month stability period are shown in Fig 1B and 1C.

*Mice Immunizations*: BALB/c mice were divided into ten dose groups of ten mice each. BALB/c mice in Group 1 were immunized with Alhydrogel alone; BALB/c mice in Group 2 were immunized with *Na*-GST-1 alone; and BALB mice in Groups 3 to 9 were immunized in 1.75-fold increasing fractional doses of the vaccine starting from 1 μg to 30 μg of *Na*-GST-1, with the ratio of the dose of *Na*-GST-1 to Alhydrogel constant at 0.125 (Table 1). Study Group 10 (6 μg of formulated reference standard *Na*-GST-1) was included as an in-house reference control (lot# 082709APM) (Table 1). All immunizations were administered by the intraperitoneal route. Fractional dosing generated the doses: i.e., a volume containing the exact amount of *Na*-GST-1/Alhydrogel drug product was withdrawn into the syringe as opposed to diluting the vaccine to achieve these doses.

**Table 1:** The design and results of the *Na*-GST-1 relative potency study.

Group	*Na*-GST-1 (μg)	Alhydrogel (μg)	Injection Volume (mL)	BALB/c Responders by Month^a^							
				N	0	12	18	24	36	48	60
1	--	240	0.30	10	0	0	0	0	0	0	0
2	30	N/A	0.02	10	0	0	0	0	0	0	0
3	30	240	0.30	10	8^b^	10	9	10	10	9	8
4	17	136	0.17	10	5	9	10	10	10	10	7
5	10	80	0.10	10	2	8	9	6	8	9	5
6	6	48	0.06	10	1	7	2	7	4	5	4
7	3	24	0.03	10	0	0	1	1	1	2	1
8	2	16	0.02	10	0	0	0	0	0	0	0
9	1	8	0.01	10	0	0	0	0	0	0	0
10^c^	6	48	0.06	10	3	9	9	5	3	7	4

^a^ The number of BALB/c mice seroconverting by dose group, with ten mice per group. Groups 1 & 2 were vaccinated with Alhydrogel 0.8 mg/mL or *Na*-GST-1 2 mg/mL of clinical drug substance alone, respectively. Groups 3–9 were vaccinated with decreasing fractional doses of the *Na*-GST-1/Alhydrogel clinical lot.

^b^ Lower due to death of one BALB/c mouse.

^c^ Immunizability group using a research standard reference lot of *Na*-GST-1/Alhydrogel.

### Murine IgG against *Na*-GST-1 measured by a qualified indirect ELISA

A qualified indirect ELISA described by Jariwala et al [15] and Bethony et al [17, 18] was used to measure levels of IgG against *Na*-GST-1 in murine serum samples. Ninety-six-well Polysorp microtiter plates (NUNC) were coated with 1 μg/mL of recombinant *Na*-GST-1 onto which experimental mouse sera were also added. Colorimetric reactions were read at a wavelength of 492nm on a SpectraMax 340PC^384^ (Molecular Devices) using SOFTmax Pro 5.4 for Windows for data capture and analysis.

### Four-parameter logistic log modeling of the Standard Calibration Curve (SCC) using a Standard Reference Serum (SRS) of murine IgG against *Na*-GST-1

**Fig 2:**
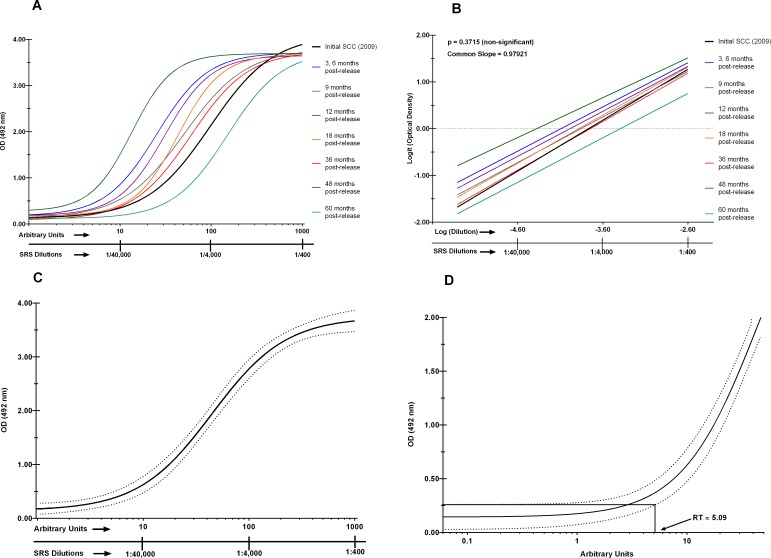
Generating a Global Standard Calibration Curve (GSCC) for Vaccine Potency Testing. **(A)** Nine standard calibration curves (SCC) generated at different time points were plotted along a four-parameter logistic log scale, where the X-axis represents the log of the dilution of SRS and the Y-axis its Optical Density (OD) of 492nm. The colored lines represent the anti-*Na*-GST-1 IgG ELISAs performed at eight time points over 60 months. **(B)** Linearization of the eight SCCs shown in (A) using a logit-log scale. Here the X-axis represents the log of the dilution and the Y-axis represents the fully specified logit of OD_492nm_. Tests of parallelism were performed using an ANOVA test, which indicated no significant departure from parallelism (p = 0.2212). **(C)** A global SCC (GSCC) with the 95% confidence interval generated by combining the eight curves shown in panel (A). **(D)** Reactivity threshold as determined from the GSCC shown in panel (C). The term OD refers to Optical Density and the term SRS refers to the Standard Reference Serum.

A standard reference serum (SRS) of murine IgG against *Na*-GST-1 was generated in 50 BALB/c mice with immunizations on Day 0 (prime) and then Day 21 (boost), with 0.05 μg *Na*-GST-1/80μg Alhydrogel co-administered with 5μg CpG 10104 delivered intramuscularly (IM). A negative control (NC) pool was similarly prepared using ten BALB/c mice immunized IM with 80μg Alhydrogel only. The SRS was serially diluted in duplicate along 11 columns of the first two rows of each ELISA plate to generate a dilution–response curve modeled into a SCC by a four-parameter logistic-log function as described by Jariwala et al [15]. The linearity and parallelism of the SCCs were assessed as part of the qualification of the SRS, with the SCCs linearized through a transformation and an Analysis of Variance (ANOVA) test [15]. The Arbitrary Units (AU) of anti-*Na*-GST-1 IgG were obtained as described by us in Jariwala et al [15]. Indirect ELISAs for these studies were performed at eight different time points post release of the new vaccine lot, with a global SCC (GSCC) established at each time point (Fig 2). The GSCC is assembled from the individual SCCs from each plate in the experimental run (as described in Jariwala et al [15] and Quinn et al [19]). Briefly, all the individual SCC in an experimental run are fit to a single sigmoidal shaped 4-PL function that estimates the combined parameters into a GSCC. The GSCC is then used to interpolate the levels of IgG against *Na*-GST-1 and to determine the numbers of seroconverted animals to elucidate the ED_50_ of the drug product at each time point.

### Determination of a Reactivity Threshold (RT), the median Effective Dose (ED_50_), potency at release, and Relative Potency (RP)

The value of the Reactivity Threshold (RT) was obtained from the GSCC as discussed by us in detail in Jariwala et al [15] (Table 1). The *probit* function of SAS 9.2 was used to estimate the ED_50_ with its 95% Fiducial Limits (95% FLs) as also described in [15] from the number of seropositive animals in each vaccination group. Based upon this model, the specification (as required by the Investigation New Drug [IND] application to the United States Food and Drug Administration (US-FDA)) for potency testing at release of the newly manufactured lot of *Na*-GST-1/Alhydrogel clinical drug product was set as the elicitation of an ED_50_ with 95% FLs from *Na*-GST-1 absorbed to Alhydrogel immunized into nine groups of ten BALB/c mice with doses ranging from 1 to 30 μg of *Na*-GST-1.

The relative potency (RP) of the clinical vaccine lot was estimated at eight post-release time points by the ED_50_ and 95% FLs, which made it possible to estimate an RP at each of these time points post release to determine the stability of the vaccine [20]. Subsequent to this evaluation, the RP and its 95% FLs are estimated by regressing the dose-response curves and then dividing by their common slope. The RP is calculated by comparing the ED_50_ at each post manufacture stability testing time point to the ED_50_ at release (T = 0 months) by the following expression:

$$M_{T}=\frac{\left(a_{T}-a_{S}\right)}{b}$$

*M*_*T*_ = log(potency ratio)
*a*_*T*_ = intersection of linear regression of responses on log(dose) from the time point being tested
*a*_*S*_ = intersection of linear regression of responses on log(dose) from the original release time point
*b* = common slope

The antilog of *M*_*T*_ is the ratio between the release potency time point (*a*_*S*_) and then a time point subsequent to release (*a*_*T*_) of the clinical drug product. All data are deposited in the Dryad Digital Data Repository (doi:10.5061/dryad.72v34) from the Dryad data repository. [21].

### Desorption of *Na*-GST-1 from Alhydrogel

*Na*-GST-1/Alhydrogel was treated with a sodium citrate buffer to desorb the recombinant protein from Alhydrogel. In brief, a solution of 100 mM sodium citrate, 92 mM dibasic sodium phosphate, pH 8.9 (unadjusted) was prepared and sterile filtered. The desorption buffer was then mixed with the re-suspended formulation at a ratio of 2:1 (buffer: vaccine), inverted ten times, and incubated for 60 minutes at room temperature (mixing by inversion every 20 minutes). The mixture was then centrifuged at 2,000 g for two minutes and supernatant (desorbed protein solution) or pellet (Alhydrogel) mixed with 2X sample buffer and analyzed via SDS-PAGE. Methodology for determining vaccine identity and integrity by SDS-PAGE including densitometry are reported in Plieskatt et al [22]. Pre-cast 4–20% Tris-glycine gels (Invitrogen) were utilized for electrophoresis under reduced (DTT) and non-reduced conditions, followed by Coomassie staining utilizing Phastgel Blue R (GE Healthcare) or silver staining [22]. Densitometry was completed using a GS-800 calibrated densitometer (Bio-Rad) with Quantity One (Bio-Rad) Software for analysis.

### Fractionation of IgG from sera of animals and humans immunized with *Na*-GST-1/Alhydrogel

Mouse serum samples obtained from dose-ranging studies of *Na*-GST-1/Alhydrogel were used to optimize the assay. In brief, IgG were fractionated from Study Day 42 sera of mice immunized with fractional doses of approximately 6, 10, 17, and 30 μg in a prime-boost regimen. Human plasma samples were utilized from two separate Phase 1 studies of the safety and immunogenicity of *Na*-GST-1/Alhydrogel in non-endemic areas (Washington, DC [NCT01385189] and Belo Horizonte, Brazil [NCT01261130]) and from a *N*. *americanus* endemic area in Minas Gerais state, Brazil (NCT01261130). In each trial, subjects were vaccinated on Study Days 0, 56, and 112, with blood collected on Study Day 126 (i.e., two weeks after the third vaccination) for the primary assessment of immunogenicity. Plasma collected prior to prime immunization (Study Day 0) and two weeks after the third immunization (Study Day 126) were IgG fractionated for the purposes of the neutralization assay described below. In the United States Phase 1 trial, 34 of 40 enrolled subjects completed the Study Day 126 visit, whereas in the Brazil trial, 96 of 102 enrolled subjects did.

IgG fractions from human plasma and mouse serum samples were purified by Protein G Spin Plates (Thermo Scientific) according to the manufacturer’s protocol. In brief, Protein G Spin Plates were equilibrated to room temperature and equilibrated with Binding Buffer (0.1M sodium phosphate, 0.15M sodium chloride, pH 7.2). All sera or plasma samples were diluted 1:1 in Binding Buffer, added to the wells of the spin plates, and the spin plates agitated on a plate shaker for 30 minutes at RT. Protein G Spin Plates were centrifuged with collection plates to remove the flow-through. Four wash steps consisted of the addition of binding buffer to each well followed by centrifugation of the spin plates with collection plates. To elute the total IgG from sera or plasma sample, 0.1M glycine pH 2.0 was added to each well followed by agitation on a plate shaker for one minute. The spin plates were centrifuged to collect the elution into collection plates already containing 1M Tris pH 9.0 to neutralize the pH of the eluate. Purified IgG was stored at 2–8°C until analysis within seven days.

### The *Na*-GST-1 neutralization assay

A schematic of the neutralization assay is shown in Supplementary S1 Fig. In brief, neutralization of *Na*-GST-1 activity by purified IgG from mice or humans immunized with *Na*-GST-1/Alhydrogel was measured utilizing the GST Fluorometric Activity Assay Kit (BioVision). To assay the purified human IgG, the manufacturer’s protocol was followed with modifications to sample volumes and as further noted here. In a 96-well black plate (Thermo Scientific), duplicate sample wells were prepared with a total volume of 100 μl, consisting of 90 μl of *Na*-GST-1 (0.25 μg) diluted in GST Assay Buffer and 10 μl of purified IgG. Blank wells contained 100 μl of GST Assay Buffer alone. Additional control wells (total volume of 100 μl) contained GST alone or IgG and were measured in quadruplicate. Plates were incubated at 37°C for one hour with agitation. After incubation, 10 μl of Glutathione was added to each well. To initiate the reaction, monochlorobimane solution was diluted 1:50 in GST Assay Buffer and 100 μl was added to each well. The plate was placed in the SpectraMax Paradigm Multimode Plate Reader (Molecular Devices), shaken for one second, and then fluorescence measured at Ex/EM 380 nm/460 nm every five minutes for one hour. The measurement of Relative Fluorescence Units (RFUs) at 15 minutes was used for analysis. Purified mouse IgG was assayed in the same manner except for an experiment where the volume of purified IgG was varied at 1, 5, 10, and 20 μl.

### Western blots for *Na*-GST-1 specific IgG using sera of animals and plasma of humans immunized with *Na*-GST-1/Alhydrogel

To confirm the presence of *Na*-GST-1 specific IgG in the purified mouse and human IgG samples, Western Blots were performed. Five micrograms of reduced and non-reduced recombinant *Na*-GST-1 were diluted in Sample Buffer (Life Technologies) and heated at 95°C for five minutes and loaded on 4–20% Tris-glycine gels (Life Technologies) with See Blue Plus2 standard (Life Technologies). For reduced *Na*-GST-1 samples, β-mercaptoethanol was included in the sample preparation. The gels were run in SDS running buffer (Life Technologies) at 135V for 90 minutes using the PowerEase 500 Power Supply and XCell SureLock Mini-Cell system (Invitrogen). Gels were transferred to nitrocellulose membranes (Life Technologies) in Transfer Buffer (Life Technologies) at 30V for 60 minutes using the PowerEase 500 Power Supply and XCell II Blot Module (Life Technologies) and stained with Ponceau S (Sigma-Aldrich) to confirm transfer of the protein occurred.

#### Anti-*Na*-GST-1 IgG (mouse)

Blocking, primary antibody incubation, washes, secondary antibody incubation, and developing were performed with the WesternBreeze Chromogenic Immunodetection System (Life Technologies) according to the manufacturer’s protocol. The purified mouse IgG (primary antibody) was diluted 1:200 in supplied buffer.

#### Anti-*Na*-GST-1 IgG (human)

Following Ponceau S staining, membranes were blocked in PBS with 0.05% Tween 20(PBS-T) and 3% skim milk powder for 1 hour (room temperature) or overnight (2–8°C). Membranes were then incubated with purified human IgG diluted 1:200 in blocking buffer for 45 minutes with gentle shaking at room temperature. Following three washes with PBS-T, the membranes were then incubated with the secondary antibody anti-human total IgG (KPL) diluted at 1:10,000. Membranes were again washed three times with PBS-T, and developed with BCIP/NBT Phosphatase Substrate System (KPL).

## Results

### Modeling the standard calibration curve and determining its linearity and a reactivity threshold

**Fig 3:**
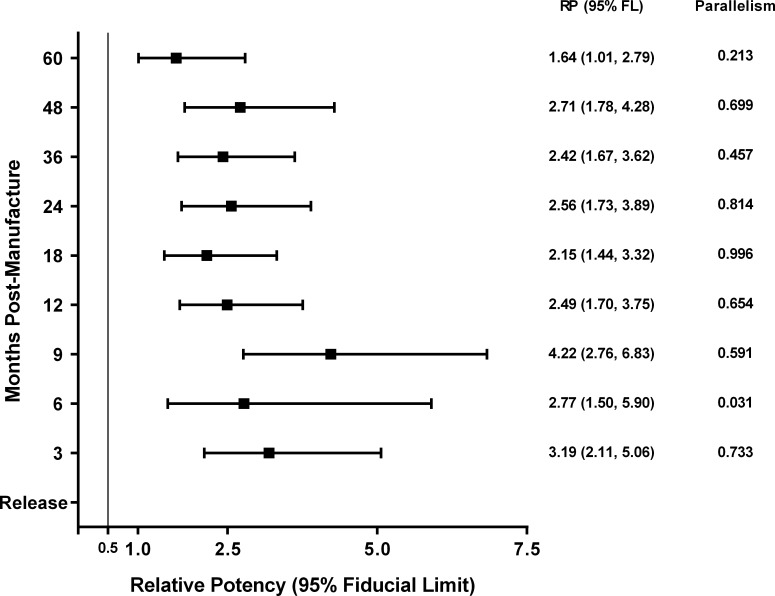
Relative Potency of *Na*-GST-1/Alhydrogel. Relative Potency (RP) and the 95% Fiducial Limits of *Na*-GST-1/Alhydrogel lot #09-69F-001 at the time of release (0 months) and at post-manufacture testing time points of three months through 60 months of storage. The specification for acceptance was that the upper 95% Fiducial Limit of the RP should not be less than 0.50.

Following pilot potency assay development as described in by us in [15] and further outlined in the bioassay study design (Fig 1), the reactivity threshold (RT) was determined for each time point during the 60 months of stability testing. Fig 2A shows the sigmoidal 4-parameter logistic log (4-PL) function of the SCC for the potency studies of *Na*-GST-1/Alhydrogel as described previously by us [15]. The parallelism of the linearized SCCs (N = 9), along with the p-values from the ANOVA test are shown in Fig 2B and Fig 3, with the ANOVA test showing no significant deviation from parallelism for the nine SCCs except at the six-month time point (p = 0.031) (Fig 3). Fig 2 Panel C shows the most recent (60-month) 4-PL global SCC (95% CIs).

### Determination of the ED_50_ using a *probit* model

The number of responders by dose group is shown in Table 1, which includes results from potency testing at lot release and at nine subsequent time points post-release (60 months). The ED_50_ at lot release was 15.74 μg (95% FL = 11.74, 23.36) from which the RP specification for the manufactured lot of *Na*-GST-1/Alhydrogel was established for subsequent stability testing time points. The specification for potency in the Investigational New Drug (IND) application to the US Food and Drug Administration for *Na*-GST-1/Alhydrogel requires that the upper 95% FL of the RP value remains above 0.50 (Fig 3): i.e., a point that describes a vaccine potency that is half of the potency at the time of release. In other words, if the value of the upper 95% FL falls below the value of 0.50, the vaccine lot of *Na*-GST-1/Alhydrogel is considered to have “lost” its potency, which means that this lot of vaccine could no longer be used in clinical trials with human volunteers. However, increased potency was observed after three months of storage post-manufacture, with the RP 3.19-times higher than the value obtained at lot release (Fig 3). At subsequent testing time points, the RP remained stable at this value over the remaining 60 months of potency testing (Table 2, Fig 3).

**Table 2:** The median Effective Dose 50 (ED_50_) for Potency at Lot Release and Relative Potency over 60 months for the *Na*-GST-1/Alhydrogel Hookworm Vaccine.

	Months Post Manufacture						
Potency measure	0^a^	12	18	24	36	48	60
ED_50_^b^	15.74	6.26	7.31	6.12	6.39	5.88	10.44
(95% FL^c^)	(11.74, 23.36)	(4.74, 8.28)	(2.97,16.10)	(4.56, 8.10)	(4.87, 8.33)	(2.56, 12.17)	(7.21, 16.23)
RP ^d^	--	2.49	2.15	2.56	2.42	2.71	1.64
(95% FL)	--	(1.70, 3.75)	(1.44, 3.32)	(1.73, 3.89)	(1.67, 3.62)	(1.78, 4.28)	(1.01, 2.79)
Parallelism^e^	--	0.654	0.996	0.814	0.457	0.699	0.213

^a^ Potency at “lot release” as it is the first time point after current Good Manufacturing Practice (cGMP) manufacture.

^b^ Effective Dose 50 or the lowest concentration of the vaccine that seroconverts 50% of the animals in a defined dose group of ten animals.

^c^ FL refers to the 95% Fiducial Limits.

^d^ RP refers to Relative Potency

^e^ Chi-square p-value. Parallelism for Relative Potency degrees freedom (df) = 10.

### Decrease in desorption efficiency of *Na*-GST-1 from Alhydrogel post manufacture coincides with a gain in relative potency

**Fig 4:**
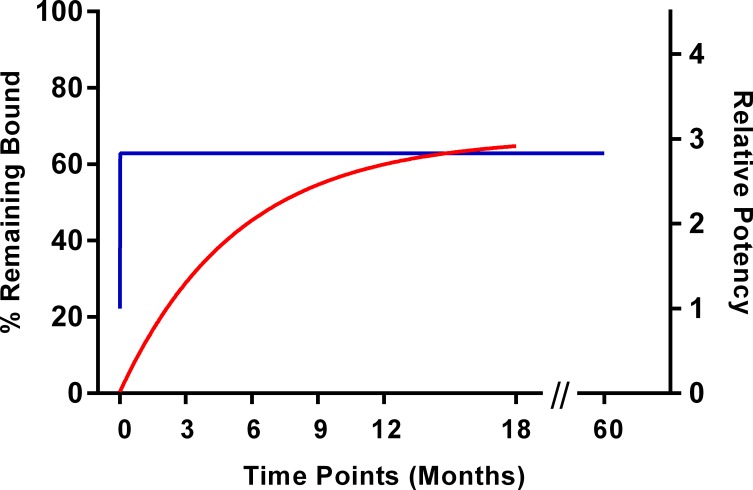
Association between the percentage (%) of *Na*-GST-1 remaining bound to Alhydrogel following desorption with Relative Potency (RP) through 18 months post manufacture. At time t = 0, *Na*-GST-1 could be completely removed from the Alhydrogel following treatment with sodium citrate. A gain in potency as represented by an increase in RP occurred during the initial three months post manufacture of lot #09-69F-001 of *Na*-GST-1/Alhydrogel. Also, the ability to desorb the *Na*-GST-1 from Alhydrogel decreased with time and plateaued by nine months post manufacture. A one-phase exponential decay model is used to graphically represent this shift. Left Y-axis: Percentage *Na*-GST-1 remaining bound to Alhydrogel after desorption treatment with sodium citrate. Right Y-axis: RP of *Na*-GST-1/Alhydrogel clinical drug product as measured by the potency assay over time through eighteen months post manufacture. The X-axis refers to the time points post manufacture.

The change in relative potency during the first three months post manufacture of *Na*-GST-1 to Alhydrogel could be attributed to the increased affinity of *Na*-GST-1 to Alhydrogel during the first three months of the storage as shown represented in Fig 4, which demonstrates the relationship between the RP and the percentage of protein remaining bound to Alhydrogel after desorption conducted at the same time points.

The *Na*-GST-1 vaccine was formulated as a suspension of 0.1 mg/mL *Na*-GST-1 and 0.8 mg/mL Alhydrogel and was optimized in a glucose/imidazole buffer to permit 100% absorption of the recombinant protein to Alhydrogel as well as to enhance stability over time [22]. The clinical lot of *Na*-GST-1/Alhydrogel met other biochemical specifications at release, including 100% absorption to Alhydrogel, identity as *Na*-GST-1, and purity by SDS-PAGE [16]. *Na*-GST-1/Alhydrogel demonstrated biochemical stability as indicated by this series of stability assays conducted over time, including identity and purity, and remained consistently absorbed to Alhydrogel [16]. In addition to this routine stability testing, the cGMP lot of *Na*-GST-1/Alhydrogel vaccine was subjected to testing including isoelectric focusing (deamidation) and antigen desorption, with no changes observed in the isoelectric point (which would indicate deamidation) with these data shown in [22]. A sodium citrate buffer (pH 8.9) was utilized to desorb the recombinant protein *Na*-GST-1 from its adjuvant Alhydrogel. The efficiency of this desorption (i.e., the ability to desorb *Na*-GST-1 from Alhydrogel) as measured by SDS-PAGE and densitometry, decreased over the first nine months post manufacture but stabilized thereafter (Fig 4). When plotting the RP of *Na*-GST-1/Alhydrogel versus the desorption efficiency of *Na*-GST-1/Alhydrogel (Fig 4), this association is clearly apparent.

### Western blotting for IgG against *Na*-GST-1 from mice and humans immunized with *Na*-GST-1/Alhydrogel

**Fig 5:**
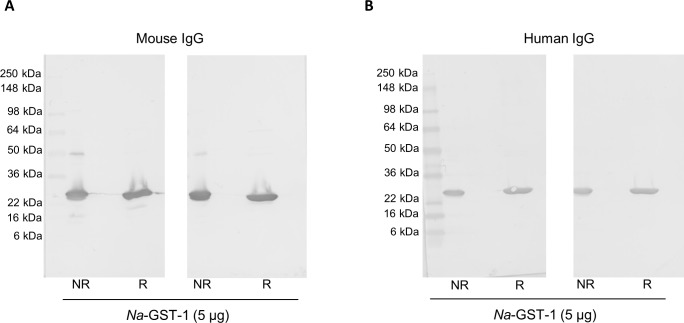
Western Blot analyses using purified IgG. *Na*-GST-1 was run under non-reducing (NR) and reducing (R) conditions and analyzed via Western blots to confirm reactivity of purified IgG obtained from mouse and human samples. **(A)** Western blot results from mouse IgG purified from two (Left and Right blots) random serum samples obtained from animals immunized with *Na*-GST-1/Alhydrogel; **(B)** Western blot results from human IgG purified from two (Left and Right blots) plasma samples obtained from clinical trial subjects immunized with *Na*-GST-1/Alhydrogel.

Antibodies (IgG) purified from both mouse sera and human plasma were confirmed to be reactive to the recombinant antigen *Na*-GST-1. Western blots (Fig 5) performed on sera from a selection of BALB/c mice immunized with *Na*-GST-1/Alhydrogel and from a selection of human plasma from a Phase 1 clinical trial of *Na*-GST-1/Alhydrogel in Brazilian adults confirmed the presence of IgG against *Na*-GST-1 in these purified samples (Fig 5). Specifically, these purified IgG samples recognized both non-reduced and reduced *Na*-GST-1 at approximately 24 kilo Daltons (kDa). It should be noted that *Na*-GST-1 under non-reduced conditions appears as a doublet as previously reported [16, 22].

### Inhibition of catalytic activity of *Na*-GST-1 by IgG from mice immunized with *Na*-GST-1/Alhydrogel

Having demonstrated the ability to purify IgG from mouse sera and the positive reactivity of such sera with recombinant *Na*-GST-1, we employed analysis with a fluorescence-based assay to measure possible inhibition of such antibodies to *Na*-GST-1 catalytic activity. It should be noted that this assay measured Glutathione-S-Transferase activity, and therefore is only representative of *Na*-GST-1 activity; inhibition through this assay is not necessarily indicative of the mechanism of native *Na*-GST-1 in *N*. *americanus*, which is thought to be detoxification of free heme that is the end product of the worm’s hemoglobin digestion pathway [4]. This shows that immunization with *Na*-GST-1/Alhydrogel can elicit antibodies that block the recombinant protein’s catalytic activity.

**Fig 6:**
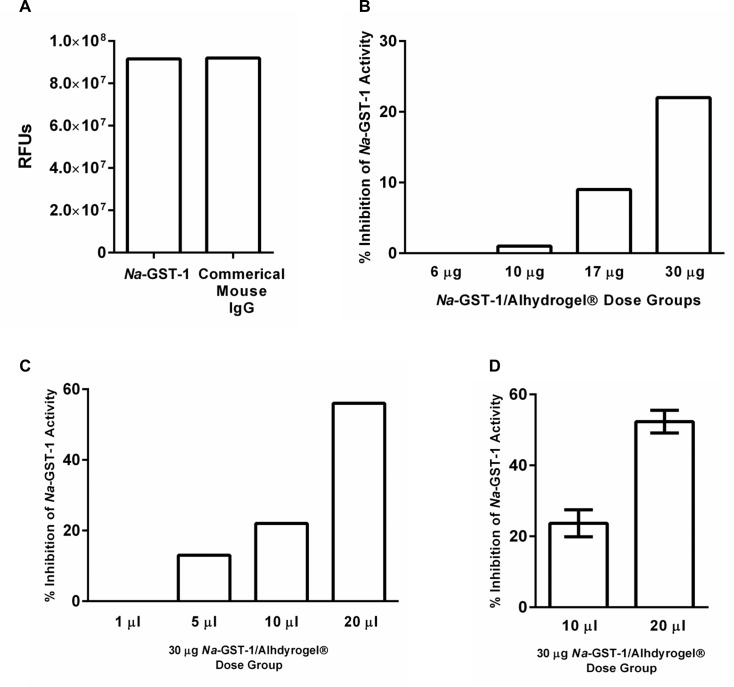
*Na*-GST-1 neutralization assay results using mouse IgG. **(A)** *Na*-GST-1 alone and *Na*-GST-1 with commercially obtained mouse IgG (not GST reactive) showed no interference or signal inhibition in the fluorescence-based assay. Fluorescence is measured in relative fluorescence units (RFUs): Y-axis. **(B)** Percent inhibition (Y-Axis) of *Na*-GST-1 catalytic activity as measured by the decrease in RFUs. X-axis shows four groups of purified IgG obtained from mice immunized with increasing doses of *Na*-GST-1/Alhydrogel (6, 10, 17, 30 μg of *Na*-GST-1/Alhydrogel). **(C)** Percentage of signal inhibition as reported by *Na*-GST-1 catalytic activity (Y-axis) with increasing amounts (volume of microliters) of purified IgG (X-axis) used in the assay from a single IgG pool purified from mice immunized with 30 μg *Na*-GST-1/Alhydrogel. **(D)** Two volumes of purified IgG (as tested in Panel C) were analyzed in three technical replicates to show reproducibility of percent inhibition of *Na*-GST-1 catalytic activity using pooled sera. The box and whiskers represent the 95% Confidence Interval (CI) of the technical replicates at each dose.

First, the non-specific activity present in IgG purified from mouse sera was measured. A commercial mouse IgG (Jackson ImmunoResearch), which is presumably non-reactive to GST, did not inhibit *Na*-GST-1 glutathione-S-transferase activity (Fig 6, Panel A). To determine if IgG purified from mice immunized with *Na*-GST-1/Alhydrogel showed a dose-dependent inhibition of *Na*-GST-1 glutathione-S-transferase activity, sera from mice immunized with increasing doses of *Na*-GST-1/Alhydrogel (Fig 6 Panel B) were assayed. With increasing doses of *Na*-GST-1/Alhydrogel, a concomitant increase in the percent of *Na*-GST-1 inhibited was observed (Fig 6 Panel B); specifically, sera pooled from mice immunized with doses of approximately 6, 10, 17, and 30 μg of *Na*-GST-1/Alhydrogel yielded purified IgG that inhibited *Na*-GST-1 glutathione-S-transferase activity by 0, 1, 9, and 22%, respectively, utilizing a consistent volume of IgG (10 μL). To determine the impact of various volumes of IgG and if a “dose response” (volume) could be observed, a purified IgG pool of sera from mice immunized with 30 μg of *Na*-GST-1/Alhydrogel was added to the reaction. In this case, inhibition of *Na*-GST-1 was 0, 13, 22, and 56% when 1, 5, 10, and 20 μl of this pool were added (Fig 6, Panel C). Finally, Fig 6 Panel D shows the results of a reference pool of purified IgG from mice immunized with 30 μg of *Na*-GST-1 run in three technical replicates in the neutralization assay to determine the reproducibility of the assay and the effect of different volumes of this reference pool,10 μl of the purified IgG pool inhibited *Na*-GST-1 activity by 24 ± 4% and 20 μl of the purified IgG pool inhibited activity by 52 ± 3%.

### Inhibition of the catalytic activity of *Na*-GST-1 by purified IgG from humans immunized with *Na*-GST-1/Alhydrogel hookworm vaccine

After demonstrating successful inhibition of *Na*-GST-1 in our enzymatic assay with sera derived from immunized mice, we sought to demonstrate the same inhibitory capability with human plasma samples obtained from Phase 1 trials of *Na*-GST-1/Alhydrogel conducted in the US and Brazil. As human plasma contains components capable of non-specific inhibition of *Na*-GST-1 and interferes in the fluorescence-based enzymatic assay. IgG was fractionated from human plasma and used alongside a commercially available purified human IgG as a control, as neither induced non-specific inhibition of *Na*-GST-1.

**Fig 7:**
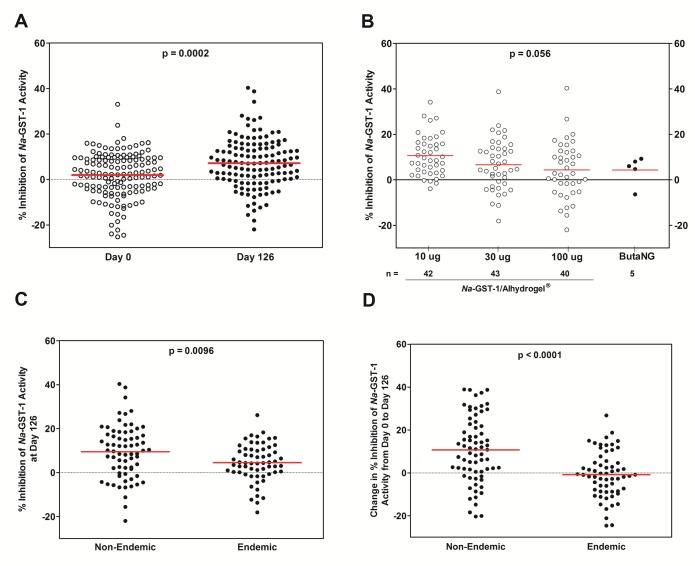
Percent inhibition of *Na*-GST-1 activity as obtained by the neutralization assay. **(A)** 142 individuals at Study Day 0 and 130 individuals at Study Day 126 (two weeks following third immunization). A statistically significant difference in inhibition was seen between the two groups (Mann-Whitney U test, p-value = 0.0002). The red line indicates the geometric mean of each group. **(B)** Various dose administrations of *Na*-GST-1/Alhydrogel (X-axis) and number of individuals (n) for each group analyzed. No statistically significant difference in inhibition was seen among the four groups tested (Kruskal-Wallis test, p-value > 0.05). The red line indicates the geometric mean of the groups. **(C)** Antibodies at study Day 126 Study, and **(D)** a comparison in antibody change from Study Day 0 to Day 126 of antibodies from individuals in hookworm non-endemic areas (USA and Belo Horizonte, Brazil) compared to individuals from a hookworm endemic area of Brazil. Statistically significant differences in inhibition and the change in inhibition were seen between hookworm-endemic and the hookworm non-endemic groups (Mann-Whitney U test, p = 0.0096, p < 0.0001; respectively). The red line indicates the geometric means of the groups.

The percent inhibition of *Na*-GST-1 glutathione-S-transferase activity was significantly greater when incubated with IgG purified from the plasma of humans immunized with *Na*-GST-1/Alhydrogel (any dose) at Study Day 126 (2 weeks after the third immunization) than when incubated with IgG purified from the plasma of humans at Study Day 0 (prior to immunization) (p-value = 0.0002) (Fig 7, Panel A) or with study controls (p-value = 0.0002). Of the 130 individuals with plasma samples at Study Day 126, only 6 samples from the United States trial and 8 samples from Brazilian yielded purified IgG capable of 20% or greater inhibition of *Na*-GST-1. The relationship between percent inhibition of *Na*-GST-1 glutathione-S-transferase activity and the dose of *Na*-GST-1/Alhydrogel administered to individuals is shown in Fig 7, Panel B. In most cases, individuals immunized with *Na*-GST-1/Alhydrogel had higher inhibition of *Na*-GST-1 glutathione-S-transferase activity than individuals who received the hepatitis B comparator vaccine (ButaNG, Instituto Butantan, São Paulo, Brazil) (Fig 7, Panel B). However, the dose of *Na*-GST-1/Alhydrogel or the co-administration of *Na*-GST-1/Alhydrogel with an aqueous formulation of the immunostimulant Glucopyranosyl-Lipid A (GLA-AF) were not found to be factors in the inhibition of the catalytic activity of *Na*-GST-1 by purified IgG from human plasma. On the other hand, purified IgG from plasma of individuals resident in hookworm non-endemic areas (USA and Belo Horizonte, Brazil) was significantly higher than the inhibition of *Na*-GST-1 glutathione-S-transferase activity compared to individuals from a hookworm endemic area in Brazil (Fig 7, Panels C and D) (p = 0.0096) and had significantly greater increases in inhibition from Day 0 to Day 126 (p < 0.0001).

## Discussion

The current manuscript focuses on a novel vaccine potency program for the *Na*-GST-1/Alhydrogel Hookworm Vaccine, with ramifications for the development of other NTD vaccines. In contrast to conventional vaccine potency testing programs, our program is unique in that if the vaccine antigen is presented to an animal immune system in a consistent form and quantity, it will induce a reproducible level of antibody responses to indicate potency at lot release (immediately cGMP post-manufacture) and then relative potency at pre-determined time points post-manufacture [11–14]. We also describe the development of a functional assay that measures the ability of IgG antibodies elicited by immunization with *Na*-GST-1/Alhydrogel to impair the catalytic activity of *Na*-GST-1 as part of the potency testing program, with the potential for it to be developed into a correlate of protection (CoP) as the vaccine advances through clinical development [23].

The first cGMP-manufactured lot of the *Na*-GST-1/Alhydrogel Hookworm Vaccine was determined to be “potent” immediately after manufacture (i.e., lot release) and remained potent over 60 months of storage at 2–8°C. A notable increase in relative potency was observed over the first nine months post release. A factor often implicated in increased vaccine potency immediately after manufacture is the vaccine’s formulation: i.e., a change in the interaction between the protein and the adjuvant. This change may be due to a number of factors, including the auto-extraction of impurities or enhanced binding of the recombinant protein to the adjuvant, resulting in conformational changes to the antigen such as aggregation or deamidation. While no concomitant aggregation or deamidation of the protein was observed three-months post-manufacture, we did observe increased binding of *Na*-GST-1 to Alhydrogel, as evidenced by the inability to remove the antigen from Alhydrogel through standard de-absorption procedures proximal to this potency time point. The increased binding may have resulted in the exposure of more immunogenic epitopes on the surface of the formulation, making the vaccine more potent in the animals. This coincidence of immunological and biochemical measures illustrates the importance of combining such assays when measuring the stability of recombinant proteins absorbed to Alhydrogel.

Our immunogenicity-based potency assay program was complemented with an assay that attempted to parallel one potential mechanism for the *Na*-GST-1 Human Hookworm Vaccine in humans [23–25]. *Na*-GST-1 has peroxidase activity as it catalyzes the conjugation of reduced glutathione to a variety of electrophiles [4–6]. As such, we designed a functional assay (S1 Fig) to measure the percent inhibition of *Na*-GST-1 glutathione-S-transferase activity when the enzyme is incubated with purified IgG from immunized animals or humans. In pools of sera from mice immunized with increasing doses of *Na*-GST-1/Alhydrogel,^,^ we observed a dose-dependent impairment of *Na*-GST-1 glutathione-S-transferase activity. Moreover, an increase in the inhibition of glutathione-S-transferase activity was also observed when the volume of purified IgG from mice was increased. A limitation of this catalytic inhibition assay is that it does not directly measure the effect of neutralizing antibodies on the putative heme detoxification role of *Na*-GST-1 in *N*. *americanus* worms. Nor does it take into account the potential effects of GSTs as immunomodulators, as shown for example by the extracellular GST from *Onchocerca volvulus* (OvGST1), which is thought to participate in the modulation of host immune responses by production of parasite-derived prostanoids that downregulate the effector response [26]. In general, plasma from study participants two weeks after the third immunization with the *Na*-GST-1/Alhydrogel Hookworm Vaccine showed a significantly higher inhibition of *Na*-GST-1 catalytic activity compared to baseline regardless of the vaccine dose or co-administration with the immunostimulant GLA-AF. However, few human samples (n = 20) produced greater than 20% inhibition of *Na*-GST-1 glutathione-S-transferase activity. This low response rate in humans indicates the need for further advances in vaccine formulation, immunization regimen, and possibly the co-administration with another immunostimulant.

Several components of the current potency assay are especially applicable to NTD vaccine production, principal among them being the choice of an animal model and the statistical methods used to measure relative potency [11, 14, 15]. Conventional potency assays use animal models based upon “their permissiveness to lethal infection with the target pathogen” [13]. However, the *Na*-GST-1/Alhydrogel potency assay measured an antibody response by dose group; hence, the choice of an animal model was not limited to models permissive to hookworm infection such as hamsters or canines [1, 27], neither of which maintain the infection long enough to measure the clinical endpoints of human hookworm disease (e.g., iron deficiency anemia) for which the vaccine will be indicated. Instead, we chose an animal model (i.e., BALB/c mice) based on the ability to induce a reproducible antibody response [15, 28]. Another critical choice was the immunization schedule (0 and 28 days), which depended on several factors, including the animal model, the route of immunization (IP), and the volume of the immunization [15]).

A final novel component to this assay was the statistical approach to determine potency and relative potency. The *Na*-GST-1/Alhydrogel potency testing program varied traditional potency assays by utilizing a ‘quantal’ assay design [2, 14, 29], which contrasts with conventional “quantitative” immunogenicity methods that use *‘measures of central tendency’* to characterize potency such as the mean, median, and standard deviation of an antibody response [11]. As noted by Giersing et al. [2] for potency testing of recombinant malaria vaccines, quantitative potency approaches have the advantage of producing readily analyzable data, but their utility is offset by the poor precision of *measures of central tendency* due to the a high degree of variation in ELISA assays.

Finally, by modifying a commercially available enzymatic assay into a functional assay for this vaccine, we were able to demonstrate that antibodies raised to *Na*-GST-1 may also have a functional application. However, further work is needed to optimize such this functional assay before it can become either a part of the current potency testing program or as a correlates of protection in the future.

## Supporting information

S1 Fig Schematic of the process for the *Na*-GST-1 catalytic inhibition assay. Outset box shows the chemical reaction of monochlorobimane (MCB) with glutathione resulting in fluorescence.
(TIF)
