# Supplementary figures and images for: Advances in neglected tropical disease vaccines: Developing relative potency and functional assays for the Na-GST-1/Alhydrogel hookworm vaccine
Source: PLoS Negl Trop Dis. 2017 Feb 13;11(2):e0005385. doi: 10.1371/journal.pntd.0005385 (PMC5325600; doi:10.1371/journal.pntd.0005385)

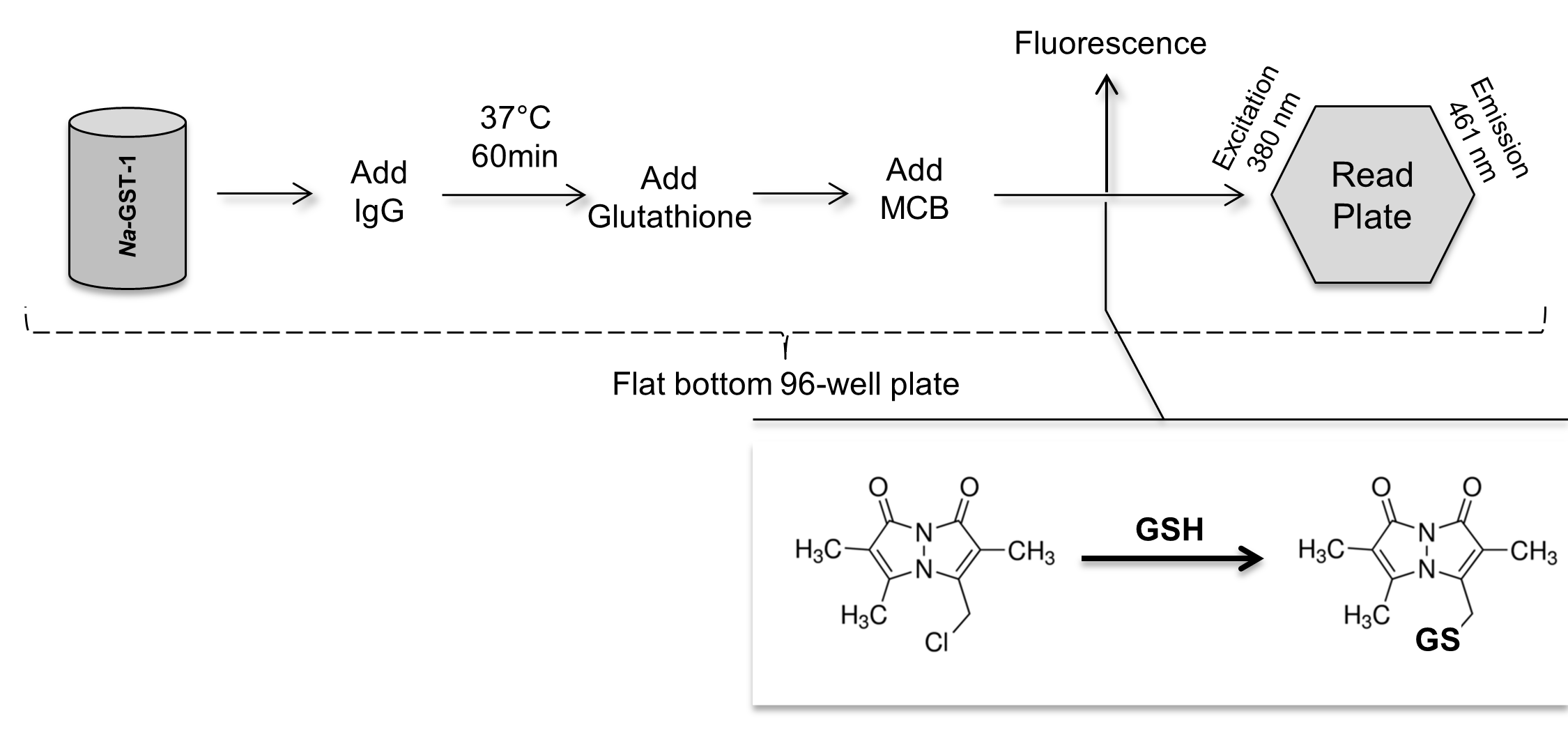

Supplement: S1 Fig — Outset box shows the chemical reaction of monochlorobimane (MCB) with glutathione resulting in fluorescence. (TIF) [file pntd.0005385.s001.tif]
